# Physiological characteristics, dry matter, and active component accumulation patterns of *Changium smyrnioides* in response to a light intensity gradient

**DOI:** 10.1080/13880209.2016.1263345

**Published:** 2016-12-09

**Authors:** Chang-lin Wang, Qiao-sheng Guo, Zai-biao Zhu, Bo-xing Cheng

**Affiliations:** Institute of Chinese Medicinal Materials, Nanjing Agricultural University, Nanjing, China

**Keywords:** Net photosynthetic rate, biomass, mannitol, choline, polysaccharide

## Abstract

**Context:***Changium smyrnioides* Wolff (Apiaceae) is an endangered medicinal plant with numerous pharmacological uses.

**Objective:** To investigate the effect of light intensity levels on the growth and accumulation of secondary metabolites of *C. smyrnioides*, cultivated seedlings were subjected to different relative light intensities via sun-shading.

**Materials and methods:***Changium smyrnioides* seedlings were subjected to five irradiance treatments (100, 60.54, 44.84, 31.39, and 10.56% sunlight) in glasshouse for 9 months. Enzymatic and non-enzymatic antioxidants with spectrophotometric method, photosynthetic parameters with Li-6400XT, dry matter accumulation and active component contents in the root with spectrophotometric and HPLC method were analyzed.

**Results:** With an increase in relative light intensity levels, activities of enzymatic and non-enzymatic antioxidants, and malondialdehyde (MDA) contents were increased overall, while net photosynthetic rate (*P_n_)* and dry matter accumulation patter first increased and then declined. The highest net photosynthetic rate (30.68 μmol/m^2^·s) and dry root weight (5.07 g) were achieved under 60.54% sunlight. Lower relative light intensity levels stimulated the accumulation levels of bioactive compounds in the roots so that the highest contents of mannitol (1.35%) and choline (405.58 μg/g) were recorded under 31.39% sunlight, and the highest polysaccharide content (10.80%) were achieved under 44.84% sunlight. With a decrease in the relative light intensity levels, the water-soluble component content increased first and then decreased.

**Discussion and conclusion:** The results revealed that 31.39–60.54% sunlight serve as appropriate relative light intensity conditions for cultivated *C. smyrnioides*.

## Introduction

*Changium smyrnioides* Wolff (Apiaceae), the sole representative of the monotypic genus *Changium*, is a herbaceous perennial plant, endemic to Eastern China (Qiu et al. 2004). It distributes in the eastern and central regions of the Yangtze River basin, and mainly in the northwest region of Zhejiang Province, the southwest region of Jiangsu Province, and the southeast region of Anhui Province (Pan et al. 1995). The dry roots of *C. smyrnioides*, commonly referred to as Changii Radix, are a valuable material in traditional Chinese medicine. Research work done in the recent past has shown that Changii Radix has many pharmacological uses like cough suppressant (Hu et al. 1995), immune function regulatory (Chen et al. 1999; Lu et al. 1991), strong tonic (Chen et al. 1992), antioxidant (Wu et al., 1993, 1994), antifatigue agent (Huang et al. 1996), antioxidation agent (Huang et al. 1996), antilipemic agent (Hua et al. 1994), anticoagulant (Li et al. 1998), antimutation agent (Li et al. 2007), small intestine motility promoter (Huang et al. 1994), etc. Active components of Changii Radix include polysaccharides, mannitol, choline, etc. Polysaccharides of Changii Radix can promote immune activity, stress resistance and intestinal motility and offer potential application value against cancer and infectious diseases (Wang et al. 1992, 2007; Huang et al. 1994; Chen et al. 1999). Mannitol can be used to treat diuresis, dehydration, antitussive symptoms, expectorant antiasthmatic symptoms and antioxidant symptoms and can have a significant effect on many diseases (Li & Chen 2006). Choline can prevent the deposition of fat into the liver and can regulate liver and gallbladder functioning (Li et al. 1994). The content of water-soluble components in Changii Radix was stipulated to exceed the legal limit of 20% in ‘Chinese Pharmacopoeia’ (2015 edition).

Owing to its medicinal importance, demand for Changii Radix has increased steadily in recent years. Both biological and anthropological factors have contributed to the recent decline of this species, and it is now classified as an endangered species in the Chinese Plant Red Book (Status: Vulnerable species) (Fu 1992). Considerable efforts have been made to cultivate *C. smyrnioides* in order to protect wild *C. smyrnioides* resources while simultaneously meeting the demand for Changii Radix. Nevertheless, yields of cultivated *C. smyrnioides* are generally low, and the quality is also inferior compared with the quality of wild variety. While seedling quality and fertilization experiments have identified optimal seed breeding methods and effective fertilizers (Li et al. 2006; Wang et al. 2007), light intensity regulation studies on the yield and quality of *C. smyrnioides* have not yet been conducted.

Yield and quality of harvested plant material is significantly correlated with light intensity levels (Briskin & Gawienowski 2001; Cai et al. 2009; Hou et al. 2010). Light intensity regulation methods can be employed to improve the yield and quality of field-cultivated medicinal plant. This notion is supported with the findings that suggest that with an increase in the light intensity levels within a suitable range, active components were increased in *Hypericum perforatum* L. (Guttiferae) (Briskin & Gawienowski 2001), *Erigeron breviscapus* (Vant.) Hand.-Mazz. (Compositae) (Su et al. 2006), *Mentha haplocalyx* Briq. (Labiatae), *Andrographis paniculata* (Burm. f.) Nees (Acanthaceae) and *Atropa belladonna* L. (Solanaceae) (Guo 2009). The *C. smyrnioides* is a shade-tolerant species that is naturally found in the gaps or along the edges of deciduous hardwood forests. However, cultivated *C. smyrnioides* grown under ambient irradiance and subjected to high-temperature stress (≥ 25 °C) at the start of the summer wilted 20–30 days earlier than the wild variety (Li et al. 2007). Hence, full sunlight conditions are clearly not conducive to high Changii Radix quality and yield levels. Some biochemical parameters, e.g., enzymatic and non-enzymatic protectants (superoxide dismutase, catalase, ascorbate peroxidase, ascorbate acid, free proline), chlorophyl and malondialdehyde are important parameter to evaluate the physiological characteristics of cultivated plants under different conditions. In this study, physiological characteristics, dry matter and active component accumulation features of *C. smyrnioides* cultivated under varying relative light intensities were investigated. The objectives were to identify appropriate relative light-intensity levels that can be employed to promote high-quality Changii Radix production on a commercial scale.

## Materials and methods

### Plant materials

When the dormant seedlings of *C. smyrnioides* began to emerge from the soil in autumn, the seedlings were dug up from the same cultivated population in Jurong of Jiangsu Province, China (N31.71°, E119.22°) on 15 October 2013. Species identification was performed by Professor Qiao-sheng Guo in Institute of Chinese Medicinal Materials at Nanjing Agricultural University, Nanjing, China. Voucher specimen number was NAS00020188 and was deposited at the Institute of Botany, Jiangsu province and Chinese Academy of Science. Six uniform-sized seedlings were cultured in each plastic container (25 cm in diameters × 30 cm in height) filled with well-mixed soil (humus:loam = 1:5; pH = 6.5) and then containers were shifted to a glasshouse (Temperature: Daytime 15–25 °C, Night 10–15 °C; Humidity 60–70%). Five randomly selected containers for each treatment were subjected to each of the five irradiance treatments viz., 100% sunlight (full sunlight, Control), 60.54% sunlight (L1 treatment), 44.84% sunlight (L2 treatment), 31.39% sunlight (L3 treatment) and 10.56% sunlight (L4 treatment) on 26 February 2014. These irradiance levels were imitated using layers of neutral-density screening attached to a steel frame. To ascertain light availability levels, photosynthetic photonﬂux densities (PPFD) were measured on clear days using a luminometer (LX-101, LUTRON, Taiwan) positioned 40 cm above the ground (near upper functional leaves), and relative light intensity levels were calculated. Seedlings were watered 500 mL each container once a week and were fertilized monthly with 20 g NPK compound fertilizer (AKANG, N:P_2_O_5_:K_2_O = 16:16:16, Russia) from 15 October 2013 to 15 July 2014.

### Biochemical analyses

Some upper functional leaves were cut out on 27 April 2014, rapidly frozen in liquid nitrogen and stored at −80 °C so that these could be analyzed for enzymatic and non-enzymatic protectants (superoxide dismutase, catalase, ascorbate peroxidase, ascorbate acid, free proline), chlorophyll, and malondialdehyde contents.

Frozen leaves (1 g) were homogenized with 8 mL of 0.05 M sodium phosphate buffer solution (pH 7.8) with 1% polyvivyl-pyrrolidone (PVP) and was centrifuged at 12,000 *g* at 4 °C for 15 min. The supernatant was collected for enzyme analysis.

SOD (superoxide dismutase) activity was measured following the method presented by Giannopolitis and Ries (1977), which involves spectrophotometric measurements of the inhibition levels in the photochemical reduction of nitroblue tetrazolium (NBT) at 560 nm. One unit of enzyme activity was defined as the quantity of SOD required to produce a 50% inhibition of NBT, and specific enzyme activity levels were expressed as units/g FW (fresh weight)·h. The reaction mixture contained 50 mM sodium phosphate buffer solution (pH 7.8), 750 μM NBT, 130 mM l-methionine, 100 mM ethylenediaminetetraacetic acid (EDTA) and 20 μM riboflavin. Reactions were conducted at 25 °C under light-intensity levels of approximately 300 μmol/m^2^ s over 25 min.

CAT (catalase) activity was measured using methods presented by Abassi et al. (1998), which involve measuring H_2_O_2_ extinction decline levels at a maximum absorption rate of 240 nm. The reaction mixture contained 50 mM Tris-HCl buffer solution (pH 7.0) and 50 mM H_2_O_2_. One unit of CAT was defined as the absorbance decreased 0.1 at 240 nm.

APX (ascorbate peroxidase) activity was measured as per Dalton et al. (1987). The assay depends on the decline in absorbance observed at 290 nm as ascorbate is oxidized. The reaction mixture contained sodium phosphate buffer solution (pH 7.0), 0.9 mM ascorbate, 0.3 mM disodium ethylenediaminetetraacetate dihydrate (EDTA-Na_2_) and 0.25 mM H_2_O_2_. Enzyme activity levels are expressed in terms of μM of ascorbate oxidized/g^1^ FW·h^1^.

A modified version of method proposed by Wu et al. (2006) was employed for the assay of ascorbate acid (AsA). Leaves (1 g) were extracted with 5 mL of 5% (w/v) metaphosphoric acid, and extracted material was centrifuged at 14,000 *g* for 10 min at 4 °C. The supernatant was used for the AsA assay. The AsA assay mixture contained 0.4 mL of supernatant, 75 mM phosphate buffer (pH 7.7), 10% (w/v) metaphosphoric acid, 44% (v/v) H_3_PO_4_, 4% (w/v) 2,2-bipyridyl and 3% (w/v) FeCl_3_. The final assay mixture was incubated at 37 °C for 60 min and then cooled to room temperature. The absorbance level was recorded at 525 nm.

Levels of compatible solute like proline were measured via spectrophotometric method following Zhi and Li (2005) method. Leaves (1 g) were extracted with 10 mL 3% (w/v) sulfosalicylic acid at 90 °C for 10 min. The extracted material was then cooled at room temperature and filtered into a test tube. Filtrate (2 mL) was mixed with 2 mL glacial acetic acid and 2 mL 2% (w/v) ninhydrin. The mixture was heated in boiling water for 30 min, mixed with 4 mL methylbenzene at room temperature for 5 min and centrifuged at 3000 *g* for 5 min. The absorbance level of the supernatant was recorded at 520 nm.

The lipid peroxidation was measured in terms of malondialdehyde (MDA) contents using the thiobarbituric acid (TBA) reaction method (Chen & Cao 2008).

Chlorophyll content was measured following method of Cai et al. (2004). Fresh leaves (0.2 g) were homogenized with 10 mL 96% (v/v) ethanol, a small quantity of quartz and calcium carbonate (CaCO_3_) powder. The filtrate was diluted to 25 mL with 96% (v/v) ethanol. The absorbance was recorded at 665 nm and 649 nm. The contents of chlorophyll a (Chl a) and chlorophyll b (Chl b) were determined using the following equation:

$$\begin{array}{c} \mathrm{Content}\;\mathrm{of}\;\mathrm{Chl}\;a\;\left(\mathrm{mg}/g\right)=13.{95A}_{665}-6.{88A}_{649}\\ \mathrm{Content}\;\mathrm{of}\;\mathrm{Chl}\;b\;\left(\mathrm{mg}/g\right)=24.{96A}_{649}-7.{32A}_{665} \end{array}$$

where A_665_ and A_649_ are absorption levels at 665 nm and 649 nm, respectively. After calculating the Chl a and Chl b values, total chlorophyll (Chl a + Chl b) values were determined.

### Determination of photosynthetic parameters

Photosynthetic parameters were measured using a portable photosynthesis system (Li-6400XT, LI-COR, USA) in a glasshouse from 10:00 am to 13:00 pm on 27 April 2014. Weather conditions were normal during course of investigation. Leaf area was determined using a leaf area meter (Li-3000C, LI-COR, USA). The net photosynthetic rate (*P_n_*), stomatal conductance (*G_s_*), transpiration rate (*E*) and intercellular CO_2_ concentration (*C_i_*) values were read out directly using the photosynthesis system, and *Pn/E* values were computed. Air flow rate in the gas line was recorded at 500 mL·min ^−^ ^1^. Leaf room temperatures were recorded at 25 °C. Relative humidity levels were recorded at 50%. The CO_2_ concentration was controlled at 380 ± 10 μmol/mol. The photon flux density (PFD) level was maintained at 900 μmol/m^2^·s (artificial light). The leaves to be used for measurements were adapted to the initial conditions for 5 min before being measured. Five leaves were sampled for each treatment.

### Analyses of dry matter accumulation and active component contents in the root

The roots were dug up on 15 July 2014 after the plants of each treatment had wilted. Harvested roots were weighed on a digital electric weighing balance after being dried in an oven for 72 h at 50 °C, The dried roots were then ground using a grinding mill to be able to pass through a 0.5 mm sieve in order to analyze the contents of active components.

### Dry matter accumulation assay

Dry matter biomass was measured by calculating the average dry root weight of each treatment. The specific root length (SRL) value was determined using the following equation:

$$\mathrm{SRL}\left(\mathrm{cm}\cdot g^{-1}\right)=\mathrm{dry}\;\mathrm{root}\;\mathrm{length}/\mathrm{dry}\;\mathrm{root}\;\mathrm{weight}$$

### Active components assay

#### Determination of the contents of polysaccharides, mannitol, choline and water-soluble components

A modified phenol-sulfuric acid method was employed for the polysaccharides assay following Du et al. (2005). Root powder (1 g) was extracted in circumfluence every 2 h in combination with petroleum ether, 95% ethanol and distilled water and was then dissolved in 80% ethanol for 12 h. The dried sediment was used for the polysaccharides assay. The polysaccharides assay mixture contained 0.2 mg of sediment, 12.5% (v/v) phenol, and 62.5% (v/v) H_2_SO_4_. The final mixture was left for 5 min and then heated in boiling water for 15 min and cooled to room temperature. The absorbance level was recorded at 490 nm.

Mannitol content was measured via spectrophotometric method following Li et al. (2006). Root powder (1 g) was extracted with 50 mL distilled water for 1.5 h and was then centrifuged at 3000 *g* for 15 min. The supernatant was reacted with 10-fold 0.02 mol/L KMnO_4_ at room temperature for 10 min. The reactant was mixed with 2-fold 0.1% L-rhamnose and 4-fold Nash reagent. The final mixture was incubated at 53 °C for 15 min and then cooled to room temperature. The absorbance level was recorded at 412 nm.

Choline content was measured via spectrophotometric method following Li et al. (1994). Root powder (30 g) was extracted in circumfluence with 160 mL 22% HNO_3_ water for 7 h. The filtrate was adjusted to pH 9.0 with sodium hydroxide (NaOH), mixed with superfluous methanol at 4 °C for 12 h, condensed to 150 mL, and finally mixed with 10 mL 5% ammonium reinecke to generate a pink crystal at 4 °C. The crystal was washed with 5 mL *n*-propanol and was then dissolved in 25 mL acetone. The absorbance level was recorded at 520 nm.

The content of water-soluble components was measured according to the ‘Chinese Pharmacopeia’ (2015 edition).

#### HPLC analysis of water-soluble components

Root powder (1 g) soaked in 20 mL of distilled water for 2 h, was extracted with an ultrasonic wave for 30 min and filtered into a test tube. The filtrate was used for an HPLC analysis of water-soluble components.

An Ettan-LC HPLC system (GE-Healthcare, USA) was used to perform the HPLC analysis. An HPLC fingerprint test was carried out on a C_18_ column (SHIM-PACK, 250 mm × 5.6 mm, 5 μm) at 30 °C with a sample injection volume of 100 μL. The detection wavelength was 285 nm, and the ﬂow rate was 1.0 mL·min ^−^ ^1^. A gradient elution of A (acetonitrile) and B (0.095% phosphoric acid) was used as follows: 0–13 min, 4% A, 96% B; 13–63 min, 4–25% A, 96–75% B; 63–68 min, 25–50% A, 75–50% B. The system was then restored to its initial conditions after 15 min.

The analysis method was tested according to the ‘Chinese Pharmacopeia (2015 edition) specifications. Relative standard deviation (RSD) of the relative peak area was 0.7% in the precision test, 1.2% in the stability test and 1.7% in the repeatability test.

HPLC chromatograms were compared with 20 greater peaks that were selected based on the detected peak area. Similarities between water-soluble components were calculated for the relative content using the included angle cosine method.

### Statistical analysis

Data were subjected to one-way analysis of variance (ANOVA) technique followed by Duncan’s multiple range test (DMRT) using SAS for Windows 8e (SAS Institute Inc., Cary, NC). SAS for Windows 8e was used to determine correlation coefficients.

## Results

### Activities of enzymatic antioxidants

When *C. smyrnioides* were subjected to different relative light intensity levels, significant variations in activities of enzymatic antioxidant were observed (Table 1). In similar pattern, the activities of SOD, CAT and APX increased overall with increasing light intensity. The greatest difference was observed for SOD activity. The activity of SOD recorded for full sunlight (Control) was 6 times higher than that recorded for 10.56% sunlight (L4 treatment) and 4 times higher than that recorded for 31.39% sunlight (L3 treatment). However, difference between the L3 and L4 treatments in terms of SOD activity levels were non-significant. Likewise, no significant difference was found between the L3 and L4 treatments for the activity of CAT. The lowest differences were observed for APX activity, and the highest value recorded for full sunlight (Control) was only 54% over the lowest value recorded for 10.56% sunlight (L4 treatment).

**Table 1.** SOD, APX, CAT activities and free proline, AsA, MDA content in the leaves of *C. smyrnioides* subjected to different relative light intensity: 100% sunlight (Control), 60.54% sunlight (L1), 44.84% sunlight (L2), 31.39% sunlight (L3) and 10.56% sunlight (L4).

Treatment	SOD	CAT	APX	Free proline	AsA	MDA
	(U/gFW·h)	(U/gFW·min)	(μmolVc/gFW·h)	(μg/gFW)	(μg/gFW)	(μmol/gFW)
Control	251.76 ± 6.03^a^	3.70 ± 0.21^a^	21093 ± 831^a^	261.43 ± 9.13^a^	117.31 ± 7.78^a^	4.21 ± 0.33^a^
L1	207.66 ± 6.51^b^	2.71 ± 0.18^c^	17675 ± 749^b^	220.11 ± 9.06^b^	111.78 ± 7.03^a^	3.71 ± 0.28^a^
L2	120.92 ± 4.37^c^	3.17 ± 0.18^b^	15967 ± 703^c^	180.93 ± 9.11^c^	116.13 ± 6.75^a^	3.81 ± 0.27^a^
L3	58.80 ± 3.84^d^	1.87 ± 0.11^d^	18241 ± 816^b^	140.12 ± 7.27^d^	97.54 ± 6.37^b^	3.16 ± 0.21^b^
L4	41.82 ± 3.81^d^	1.50 ± 0.08^d^	13685 ± 615^d^	80.46 ± 7.04^e^	101.08 ± 5.53^ab^	2.57 ± 0.25^c^

Different superscript letters denote significant difference at *p =* 0.05. Means ± standard deviations (SD) (*n* = 3) are shown.

### Contents of ascorbate acid, proline and MDA

As with antioxidant enzyme activities, significant differences were observed in terms of the contents of ascorbate acid, proline and MDA when *C. smyrnioides* were subjected to different relative light intensity levels (Table 1). The greatest difference was observed for proline content. With light intensity increasing, proline content increased remarkably. The highest proline content recorded for full sunlight (Control) was three times higher than the lowest one recorded for 10.56% sunlight (L4 treatment). However, no significant difference was detected in AsA contents recorded for all the treatments except L3 treatment (31.39% sunlight). Similar to the changes of proline content, MDA content increased overall with light intensity increasing. The increasing trend was significant at first, and then such an increase was no longer significant when light intensity was increased to 44.84% sunlight (L2 treatment). The highest MDA content recorded for full sunlight (Control) was 64% over the lowest one recorded for 10.56% sunlight (L4 treatment).

### Chlorophyll content

**Figure 1.**
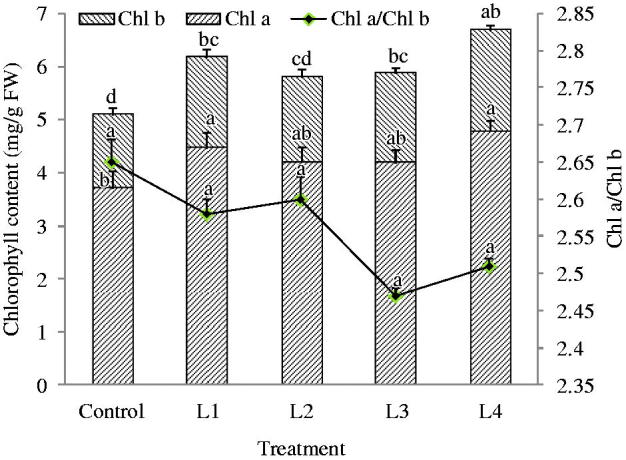
Chlorophyll content in the leaves of *C. smyrnioides* subjected to different relative light intensity: 100% sunlight (Control), 60.54% sunlight (L1), 44.84% sunlight (L2), 31.39% sunlight (L3) and 10.56% sunlight (L4). Different letters denote significant difference at *p* = 0.05. Means ± standard deviations (S.D.) (*n* = 3) are shown.

When *C. smyrnioides* were subjected to different relative light intensity levels, a significant difference in chlorophyll content was observed (Figure 1). Chl b showed more significant differences than Chl a. Variable light intensity had a pronounced influence on Chl b than on Chl a. With the exception of the control, no significant difference in chlorophyll content was observed among other treatments. The highest content of chlorophyll was detected in the L4 treatment, and the lowest one was detected in the control. The values of Chl a/Chl b were non-significant in all treatments, and that were changed from 2.3 to 3.0.

### Photosynthetic parameter changes

When *C. smyrnioides* were subjected to different relative light intensity levels, significant differences in all photosynthetic parameters except *C_i_* were observed (Table 2). The greatest degree of difference was observed for *P_n_*. With an increase in relative light intensity levels, *P_n_* first increased and then decreased. The highest value was recorded for 60.54% sunlight (L1 treatment), which was two times higher than that recorded for 10.56% sunlight (L4 treatment) and 51% over that recorded for full sunlight (Control). Meanwhile, the values of *E*, *Gs* and *Pn*/*E* recorded for 60.54% sunlight (L1 treatment) were of the highest level. This result indicates that photosynthetic capability was the best in leaves of *C. smyrnioides* cultivated under 60.54% sunlight (L1 treatment).

**Table 2.** Photosynthetic parameters in the leaves of *C. smyrnioides* subjected to different relative light intensity: 100% sunlight (Control), 60.54% sunlight (L1), 44.84% sunlight (L2), 31.39% sunlight (L3) and 10.56% sunlight (L4).

Treatment	*Pn*	*Ci*	*Gs*	*E*	*Pn*/*E*
	(μmol/m^2^·s)	(μmol/mol)	(mmol/m^2^·s)	(mmol/m^2^·s^1^)	(μmol/mmol)
Control	20.29 ± 0.12^c^	264.53 ± 1.53^a^	0.55 ± 0.03^ab^	10.11 ± 0.38^a^	1.99 ± 0.07^b^
L1	30.68 ± 2.14^a^	234.78 ± 9.89^a^	0.63 ± 0.07^a^	10.95 ± 1.15^a^	2.82 ± 0.19^a^
L2	26.22 ± 1.71^ab^	252.64 ± 5.51^a^	0.64 ± 0.05^a^	10.86 ± 1.04^a^	2.41 ± 0.14^ab^
L3	22.88 ± 2.08^bc^	253.78 ± 8.28^a^	0.54 ± 0.05^ab^	9.98 ± 0.96^a^	2.29 ± 0.11^ab^
L4	13.65 ± 1.32^d^	264.67 ± 4.18^a^	0.34 ± 0.02^b^	7.04 ± 0.71^b^	1.95 ± 0.09^b^

Means ± standard deviations (S.D.) (*n* = 5) are shown. Different superscript letters shown after means denote significant differences at *p =* 0.05.

### Dry matter accumulation

**Figure 2.**
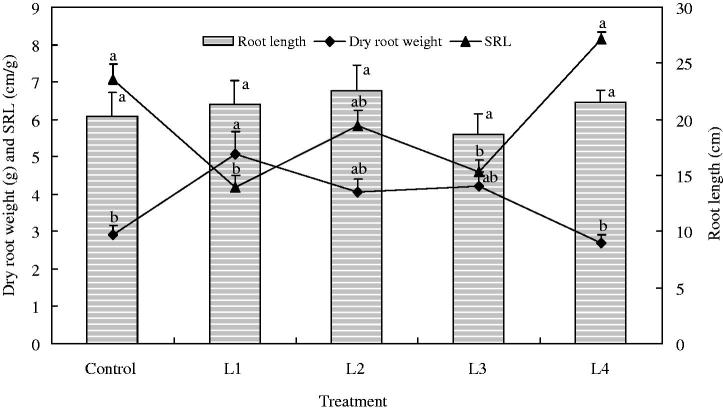
Dry matter accumulation in the roots of *C. smyrnioides* subjected to different relative light intensity: 100% sunlight (Control), 60.54% sunlight (L1), 44.84% sunlight (L2), 31.39% sunlight (L3) and 10.56% sunlight (L4). SRL = specific root length. Different letters denote significant difference at *p* = 0.05. Means ± standard deviations (S.D.) (*n* = 30) are shown.

Significant difference was also observed in dry matter accumulation when *C. smyrnioides* samples were subjected to different relative light intensity levels (Figure 2). As with the value of *P_n_* in leaves, dry matter accumulation in roots first increased and then decreased with light intensity increasing. The highest value was recorded for 60.54% sunlight (L1 treatment), which was 89% over that recorded for 10.56% sunlight (L4 treatment) and 75% over that recorded for full sunlight (Control). No significant difference in root length was found. This shows that dry matter accumulation increased mainly through root thickening. This conclusion was validated in terms of SRL level changes. SRL levels changed in the reverse pattern of that of dry matter accumulation. The lowest value was detected for 60.54% sunlight (L1 treatment).

### Contents of polysaccharides, mannitol, choline and water-soluble components

Likewise, significant differences were observed in contents of polysaccharides, mannitol and choline when *C. smyrnioides* were subjected to different relative light intensity levels (Table 3). The greatest difference was observed for mannitol content. Mannitol content increased from 0.87% to 1.35% as relative light intensity decreased from 100% to 31.39% and then decreased to 0.80% as relative light intensity continued to decrease to 10.56%. Similar to mannitol content, choline content increased overall as relative light intensity decreased from 100% to 31.39% and then deceasing as relative light intensity continued to decrease to 10.56%. Polysaccharide content increased from 6.53% to 10.80% as relative light intensity decreased from 100% to 44.84% and then decreased to 8.79% as relative light intensity decreased further to 10.56%. Conforming to the ‘Chinese Pharmacopeia I’ (2015) standards, the contents of water-soluble components exceeded the legal limit of 20% in all of the five treatments. With a decline in relative light intensity, the content of water-soluble components gradually increased. However, no significant difference was found for the five treatments.

**Table 3.** The contents of polysaccharides, mannitol, choline and water-soluble components in the roots of *C. smyrnioides* subjected to different relative light intensity: 100% sunlight (Control), 60.54% sunlight (L1), 44.84% sunlight (L2), 31.39% sunlight (L3) and 10.56% sunlight (L4).

Treatment	Mannitol	Choline	Polysaccharide	Water-soluble extract
	(%)	(μg/g)	(%)	(%)
Control	0.87 ± 0.04^d^	258.81 ± 15.98^b^	6.53 ± 0.21^b^	29.86 ± 2.13^a^
L1	1.05 ± 0.06^c^	384.63 ± 21.67^a^	8.75 ± 0.74^ab^	32.18 ± 2.22^a^
L2	1.17 ± 0.08^b^	352.51 ± 23.44^a^	10.80 ± 0.89^a^	32.43 ± 2.73^a^
L3	1.35 ± 0.09^a^	405.58 ± 27.52^a^	9.79 ± 0.60^a^	33.12 ± 2.16^a^
L4	0.80 ± 0.04^d^	243.74 ± 20.13^b^	8.79 ± 0.23^ab^	33.97 ± 2.27^a^

Different superscript letters denote significant difference at *p* = 0.05. Means ± standard deviations (S.D.) (*n* = 3) are shown.

### Correlation analyses

Positive correlations were found between antioxidant enzymes activities, ascorbate acid, proline and MDA content (Table 4). Particularly significant positive correlations were found between SOD and CAT. This may be attributed to the fact that SOD is known to be a substrate-inducible antioxidant enzyme and to the fact that CAT and SOD are closely related in the substrate and product. The higher CAT activity denoted that plants were better able to decompose H_2_O_2_ generated from SOD. As a major cytoplasmic osmoticum, free proline content was found to be significantly related to antioxidant enzyme activities. Additionally, lipid peroxidation levels (as measured in MDA content) were found to be related significantly to antioxidant enzyme activities, ascorbate acid content and proline content. This denoted a close relationship between these parameters. The contents of chlorophyll and active components were found to be mainly negatively related to antioxidant enzymes activities and the contents of ascorbate acid, proline and MDA. In particular, the content of water-soluble components, the unique criteria for determining the quality of Changii Radix in ‘Chinese Pharmacopeia I’ (2015), was found to be significantly related to other factors. However, positive correlations were found between *P_n_* levels, dry root weight and the contents of the four active components.

**Table 4.** Correlation analysis of antioxidant enzyme activities, *Pn*, free proline (Pro.), AsA, MDA, chlorophyll (Chl.), dry root weight (DRW), polysaccharides (Pol.), mannitol (Man.), choline (Cho.) and water-soluble components (WSE) contents in the roots of *C. smyrnioides* subjected to different relative light intensity.

	SOD	CAT	APX	Pro.	AsA	MDA	Chl.	Pn	DRW	Man.	Cho.	Pol.	WSE
SOD	1												
CAT	0.86^b^	1											
APX	0.75	0.68	1										
Pro.	0.96^a^	0.92^b^	0.84^b^	1									
AsA	0.82^b^	0.94^a^	0.42	0.81	1								
MDA	0.88^b^	0.97^a^	0.77	0.96^a^	0.87^b^	1							
Chl.	−0.67	−0.83^b^	−0.89^b^	−0.80	−0.59	−0.85^b^	1						
Pn	0.46	0.43	0.31	0.57	0.42	0.58	0.22	1					
DRW	0.16	0.06	0.11	0.26	0.06	0.24	0.07	0.93^a^	1				
Man.	−0.27	−0.09	0.14	−0.03	−0.26	0.07	−0.14	0.56	0.67	1			
Cho.	−0.09	−0.07	0.13	0.10	−0.18	0.12	0.00	0.79	0.91^b^	0.91^b^	1		
Pol.	−0.64	−0.36	−0.59	−0.50	−0.28	−0.33	0.42	0.30	0.46	0.67	0.60	1	
WSE	−0.91^b^	−0.91^b^	−0.87^b^	−0.93^a^	−0.77	−0.91^b^	0.89^b^	0.24	0.09	0.21	0.19	0.68	1

^a^0.01 > *p >* 0.001; ^b^0.05 > *p >* 0.01.

### HPLC analysis of water-soluble components

**Figure 3.**
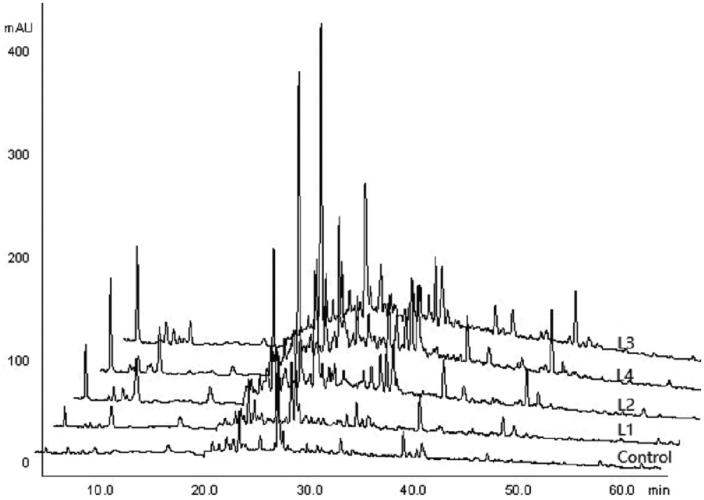
HPLC chromatograms on water-soluble components in the roots of *C. smyrnioides* subjected to different relative light intensity: 100% sunlight (Control), 60.54% sunlight (L1), 44.84% sunlight (L2), 31.39% sunlight (L3) and 10.56% sunlight (L4).

Water-soluble components in the roots of *C. smyrnioides* subjected to five light intensity gradient were analyzed by HPLC chromatographic fingerprint. Five treatments produced 11 common chromatographic peaks, but in different ratios (Figure 3; Table 5). Peak shape changed in similar ways in all of the five treatments, though peak area and relative content clearly changed in the corresponding peaks (Figure 3; Table 5). The similarity coefficient calculated from relative content ranged from 0.5735 to 0.9186 in the 27 referenced peaks (Table 6). The highest value was found between the L1 and L2 treatments, and the lowest value was found between the control and L4 treatments. The highest relative content was achieved at a retention time of 27.76 min in the control and L1 treatments, and at 23.64 min in the L3 and L4 treatments. With a decline in relative light intensity, the total peak area increased. This was consistent with water-soluble components content (Table 3). By contrast, the number of all peaks declined with a decline in relative light intensity. This denoted that the sorts of water-soluble components decreased with relative light intensity decreasing.

**Table 5.** Peak areas in water-soluble components in the roots of *C. smyrnioides* subjected to different relative light intensity: 100% sunlight (Control), 60.54% sunlight (L1), 44.84% sunlight (L2), 31.39% sunlight (L3) and 10.56% sunlight (L4).

Retention Time (min)	Peak area (mAU*min)					Retention Time (min)	Peak area (mAU*min)				
	Control	L1	L2	L3	L4		Control	L1	L2	L3	L4
6.09	–	3.21	8.46	14.94	13.85	28.17	–	12.50	29.41	–	21.69
8.99	–	–	–	4.34	3.83	28.78	3.38	3.10	4.97	5.91	–
10.77	1.88	5.96	12.34	6.09	12.74	29.26	1.43	2.87	4.82	11.32	11.31
17.76	3.14	5.04	6.00	–	–	31.08	1.61	–	4.21	4.42	6.60
19.67	1.64	–	–	–	–	32.06	1.59	2.12	5.13	10.33	11.69
21.45	2.06	2.18	–	–	4.44	33.33	–	–	–	6.98	8.92
22.35	1.65	2.63	–	–	3.93	34.29	3.24	5.03	7.50	5.18	8.01
22.88	2.68	3.55	–	–	–	34.91	–	–	8.30	11.86	13.91
23.31	2.28	–	4.16	4.54	–	35.55	–	3.60	11.08	14.36	18.53
23.64	6.20	9.09	26.89	57.19	53.37	40.33	5.28	7.44	8.31	6.16	9.89
24.23	–	4.99	6.88	10.51	–	41.59	1.70	–	–	–	–
24.91	1.85	2.39	7.17	6.74	4.01	42.11	4.64	3.88	3.94	6.23	4.70
26.61	2.82	7.68	13.66	5.49	11.78	48.37	1.64	2.93	7.68	10.82	12.79
27.76	19.05	12.56	17.85	39.04	21.75	Total	69.78	102.76	198.75	242.44	257.75
Total area of detected peaks	142.01	192.09	339.15	363.54	397.20	Total number of detected peaks	203	197	190	178	175

‘–’ Denotes that a water-soluble component does not fall within the range of the 27 peaks.

**Table 6.** Similarity coefficients of water-soluble components in the roots of *C. smyrnioides* subjected to different relative light intensity: 100% sunlight (Control), 60.54% sunlight (L1), 44.84% sunlight (L2), 31.39% sunlight (L3) and 10.56% sunlight (L4).

	Control	L1	L2	L3	L4
Control	1.0000				
L1	0.7479	1.0000			
L2	0.5964	0.9186	1.0000		
L3	0.7186	0.6896	0.7631	1.0000	
L4	0.5735	0.7762	0.8952	0.9047	1.0000

## Discussion

*Changium smyrnioides* is an early-spring herb (Wang et al. 2000). Its light saturation point occurs at 850.4 μmol/m^2^ s (Fan et al. 2003). Solar energy (*Eu*) efficiency was high in the early spring and then decreased with light intensity and temperature increasing. When temperature increased to approximately 25 °C in early May, the cultivated seedlings wilted 20–30 days earlier than wild seedlings (Li et al. 2007). Full sunlight mainly resulted in heat and drought damage to the cultivated seedlings. It is well known that high temperature and drought stress cause the formation of Active Oxygen Species (AOS) and consequently activate the expression of gene encoding antioxidant enzymes (Torres-Franklin et al. 2008). Our results showed that *C. smyrnioides* effectively protect against AOS reﬂected by low MDA content when exposed to extreme temperature and drought stress. This may be explained at least in part by the fact that there was a significant increase in leaf SOD, CAT, and APX activity levels as relative light intensity increased, suggesting the stimulation of antioxidant mechanisms for the effective removal of AOS. As greater levels of SOD activity under high temperature and drought stress were accompanied by an increase in APX activity, CAT activity, ascorbate acid content and proline content in *C. smyrnioides* leaves, it may be suggested that SOD, CAT, and APX more efficiently decompose AOS. This was in agreement with numerous previous reports published in the literature that highlight the close relationship between enhanced or constitutive antioxidant enzyme activities and increased resistance to environmental stress (Ali et al. 2005; Türkan et al. 2005; Misra & Gupta 2006; Chen & Cao 2008).

Generally speaking, the value of Chl a/Chl b is roughly three in sun plant leaves and approximately 2.3 in shade plant leaves (Wang 2000). Our results showed that this value in the *C. smyrnioides* leaves ranged from 2.3 to 3.0, approaching a value of 2.3. It may be suggested that *C. smyrnioides* belong to the shade-tolerant neutral plant family, which adapts to a larger light intensity range. This may be validated by the fact that higher net photosynthetic rate (*P_n_*) and dry root weight emerged as relative light intensity increased from 31.39% to 60.54%. However, the two values decreased significantly when plants were subjected to full sunlight and 10.56% sunlight. This may be explained by the fact that the leaf mesophyll cells were damaged under full sunlight conditions, and light intensity were deficient under 10.56% sunlight conditions. Under 60.54% relative light intensity conditions, the plants achieved maximum *P_n_* and dry root weight. This denotes that 60.54% sunlight conditions may serve as the most suitable light intensity for the yield of Changii Radix.

As predicted by the Growth/Differentiation Balance Hypothesis (GDBH), rapidly growing plants have a low secondary metabolite concentration level due to the presence of a resource-based tradeoff between primary and secondary metabolic pathways. However, moderate light limitations slow growth more than carbon assimilation, which can result in the accumulation of carbohydrates in source leaves. This may in turn increase the amount of substrate available for secondary metabolism processes (Hou et al. 2010). However, when light limitations are severe enough to depress carbon assimilation processes, secondary metabolism processes are predicted to decline due to energy and substrate concentrations associated with biosynthesis (Hale et al. 2005). In conjunction with this prediction, we found positive correlations between *P_n_* levels, dry root weight levels and levels of the four active components. Moreover, the contents of mannitol, choline and polysaccharide increased as relative light intensity decreased from 60.54% to 31.39%, and these levels decreased as relative light intensity continued to decrease to 10.56%. Secondary metabolism mechanisms are certainly intricate physiological and biochemical processes. Several works on other medicinal plants showed that secondary metabolite accumulation processes were assumed to result from physiological adaptation and that such processes protect medicinal plants from oxidative stress. The AOS may serve as a stress signal that triggers the activation of antioxidant and secondary metabolism enzymes (Munné-Bosch et al. 2001; Misra & Gupta 2006; Jaleel et al. 2007a, 2007b). This may explain why certain water-soluble components content increased with relative light intensity increasing.

It is always challenging to coordinate the relationship between medicinal material quality and yield over the course of medicinal plant cultivation. While 60.54% sunlight conditions may serve as the most suitable relative light intensity settings for Changii Radix yield formation, appropriate relative light intensity for cultivated *C. smyrnioides* should fall within a range of 31.39–60.54% sunlight to maintain yields and quality levels during the rapid growing phase of the spring and early summer. Appropriate screening which could imitate suitable irradiance levels is recommended in the cultivated *C. smyrnioides.* Some interest in the potential role of medicinal and aromatic plants in intercropping systems has arisen from the widespread trend toward the cultivation of some species with organic and sustainable methods (Carrubbaa et al. 2008). And a number of references reported the optimal shade conditions for growth and development of medicinal and aromatic plants in agroforestry systems (Carrubbaa et al. 2008; Feijó et al. 2009; Sujatha et al. 2011; Yang et al. 2013). Nevertheless, in order for such methods to be employed during *C. smyrnioides* cultivation, further studies must specify plant–plant allelopathy conditions, ascertain reasonable cultivation spaces, and corresponding water and fertilizer management procedures. These issues are currently being explored in our laboratory.

## Conclusion

Significant changes in photosynthesis, growth and active component contents were observed in *C. smyrnioides* seedlings along an experimental light intensity gradient. *C. smyrnioides* effectively protected against AOS when exposed to high light intensity stress, denoting higher plasticity levels during the physiological acclimation to light intensity. Suitable sun-shading conditions may feasibly help increase dry matter accumulation and secondary metabolite content of *C. smyrnioides*. To promote the yield and quality of Changii Radix, appropriate relative light intensity should fall within a sunlight range of 31.39–60.54% during the rapid growing phase from the spring and to the early summer.
